# Deep vs. Awake Extubation and LMA Removal in Terms of Airway Complications in Pediatric Patients Undergoing Anesthesia: A Systemic Review and Meta-Analysis

**DOI:** 10.3390/jcm7100353

**Published:** 2018-10-14

**Authors:** Chang-Hoon Koo, Sun Young Lee, Seung Hyun Chung, Jung-Hee Ryu

**Affiliations:** 1 Department of Anesthesiology & Pain medicine, Seoul National University College of Medicine, Seoul 03080, Korea; vollock9@gmail.com; 2 Department of Anesthesiology & Pain medicine, CHA Bundang Medical Center, CHA University School of Medicine, Seongnam 13496, Korea; syhahaha8787@naver.com; 3 Department of Anesthesiology & Pain medicine, Seoul National University Bundang Hospital, Seongnam 13620, Korea; anejsh@naver.com

**Keywords:** airway complications, awake extubation, deep extubation

## Abstract

The purpose of this study was to compare the incidence of airway complications between extubation under deep anesthesia (deep extubation) and extubation when fully awake (awake extubation) in pediatric patients after general anesthesia. A systematic review was performed in accordance with the Preferred Reporting Items for Systematic Reviews and Meta Analyses (PRISMA) statement standards. The review protocol was registered with the International Prospective Register of Systematic Reviews (registration number: CRD 42018090172). Electronic databases were searched, without discrimination of publication year and language, to identify all randomized controlled trials investigating airway complications following deep or awake extubation after general anesthesia. The Cochrane tool was used to assess the risk of bias of trials. Randomized trials investigating airway complications of deep extubation compared with awake extubation after general anesthesia with an endotracheal tube and laryngeal mask airway (LMA) were sought. Overall airway complications, airway obstruction, cough, desaturation, laryngospasm and breath holding were analyzed using random-effect modelling. The odds ratio was used for these incidence variables. Seventeen randomized trials were identified, and a total of 1881 pediatric patients were enrolled. The analyses indicated deep extubation reduces the risk of overall airway complications (odds ratio (OR) 0.56, 95% confidence interval (CI) 0.33–0.96, *p* = 0.04), cough (OR 0.30, 95% CI 0.12–0.72, *p* = 0.007) and desaturation (OR 0.49, 95% CI 0.25–0.95, *p* = 0.04) in children after general anesthesia. However, deep extubation increased the risk of airway obstruction compared with awake extubation (OR 3.38 CI 1.69–6.73, *p* = 0.0005). No difference was observed in the incidence of laryngospasm and breath-holding between the two groups regardless of airway device. The result of this analysis indicates that deep extubation may decrease the risk of overall airway complications including cough and desaturation but may increase airway obstruction compared with awake extubation in pediatric patients after general anesthesia. Therefore, deep extubation may be recommended in pediatric patients to minimize overall airway complications except airway obstruction and the clinicians may choose the method of extubation according to the risk of airway complications of pediatric patients.

## 1. Introduction

During general anesthesia, artificial airway devices, including endotracheal tube or laryngeal mask airways (LMA), are inserted for the maintenance of airway patency to accommodate for the decreased tone of pharyngeal and laryngeal muscles [1,2]. These devices are removed during emergence from general anesthesia. However, airway complications are most frequently observed following extubation and during the subsequent recovery period due to manipulation or intervention of the airways in light planes of anesthesia. Furthermore, pediatric patients may be vulnerable to airway complications, such as hypoxemia or laryngospasm compared to adults [3,4], since they have immature alveoli, increased dead space, and increased metabolic rate [5].

The removal of airway devices is performed in one of two ways: when patients are still in a deep anaesthetized state (deep extubation), or when in a conscious and awake state (awake extubation). There is ongoing controversy regarding the optimal timing for extubation in pediatric patients with increased risk of perioperative airway complications. Some investigators, including Archie Brain who pioneered LMA, recommend awake extubation to prevent oxygen desaturation and upper airway obstruction [6]. This camp asserted that there is a higher incidence of oxygen desaturation [7] and airway complications like upper airway obstruction [8] following early removal of LMA in pediatric patients. Conversely, others have indeed demonstrated that early removal—deep extubation—of the LMA was associated with fewer complications than awake extubation [9,10]. Some authors have found no difference in the incidence of airway complications following awake vs. deep extubation in pediatric patients. Therefore, the objective of this meta-analysis of RCTs was to compare the incidence of airway complications between deep extubation and awake extubation in pediatric patients undergoing general anesthesia.

## 2. Methods

The review was performed according to a Preferred Reporting Items for Systematic Reviews and Meta-Analyses (PRISMA) statement guidelines [11] and the predefined protocol was registered with the International Prospective Register of Systematic Reviews (registration number: CRD42018090172).

### 2.1. Data Sources and Search Strategy (Literature Search)

Randomized clinical trials (RCTs) investigating the airway complications during deep and awake extubation in pediatric patients after general anesthesia were evaluated using a predefined protocol. Two authors (C.-H.K., J.-H.R.) independently searched and retrieved relevant studies from the following electronic databases: MEDLINE, EMBASE, KoreaMed, the Cochrane Central Register of Controlled Trials (CENTRAL), Cumulative Index of Nursing and Allied Health Literature (CINAHL), Scopus, and Web of Science. The last search was run on 21 November 2017.

The medical subject headings (MeSH), text words, and controlled vocabulary terms relating to deep extubation and awake extubation were sought. Results were combined using the Boolean operator “AND” with the search terms. A specific search strategy was applied for each database which is shown in Table S1. No language restrictions were applied in search strategies and randomized trials published in the English, French, Japanese, and Korean languages were included. The publication year was not limited. The bibliographic lists of relevant articles and reviews were searched for further potentially eligible trials. Pediatric patients were considered as participants of interest.

### 2.2. Study Selection (Trial Selection and Methodological Assessment)

The title and abstract of articles identified from the literature searches were evaluated by two authors (C.-H.K., J.-H.R.) independently. The full texts of relevant reports were retrieved and those articles that met the eligibility criteria of our review were selected. Any discrepancies in the study selection were resolved by discussion between the authors. An independent third author (S.H.C.) was consulted in the event of disagreement.

### 2.3. Data Collection (Data Extraction)

Data was extracted from each study independently by two pairs authors (C.-H.K., J.-H.R.), and disagreements were resolved by discussion. If no agreement could be reached, a third author (S.H.C.) was consulted.

Extracted data included: study related data (first author, year of publication, language, study size, surgery types of the study participants); baseline patient characteristic and clinical information of the study populations (age, weight); and anesthesia related data (airway device, anesthetics, neuromuscular blockade). Data collection was performed independently by two authors (C.-H.K., J.-H.R.), and disagreements were resolved by discussion. If no agreement could be reached, a third author (S.H.C.) was consulted.

### 2.4. Methodological Quality and Risk of Bias Assessment

Two independent authors (C.-H.K., J.-H.R.) evaluated the methodological quality and risk of bias of the included studies, using the Cochrane Risk of Bias tool for assessing the risk of bias of randomized trials [12]. The Cochrane tool assesses domains, including selection bias, performance bias, detection bias, attrition bias, reporting bias, and other sources of bias and, for each individual domain, classifies studies into low, unclear, and high risk of bias. Disagreements were resolved by consensus and a third author (S.H.C.) adjusted the discrepancy if agreement could not be achieved. A risk of bias graph was constructed to present the results.

### 2.5. Outcomes Assessed

The incidence of overall airway complication was considered the primary outcome measure. ‘Overall airway complications’ is regarded as the complications reported in each study as ‘total’, ‘any’ or ‘overall’ events. The secondary outcome measures included the incidence of airway obstruction (snoring, stridor, paradoxical chest, and abdominal movement requiring the use of airway adjuncts or airway support), cough, desaturation (<95 or <90%), laryngospasm (respiratory effort without airflow despite chin lift and jaw thrust, thus requiring assisted positive pressure ventilation) and breath holding (apnea longer than five seconds).

### 2.6. Data Synthesis and Statistical Analyses (Meta-Analysis)

The primary and secondary outcome measures in the present study were dichotomous variables; therefore, we (C.-H.K., S.Y.L.) calculated the odds ratio (OR) as the summary measure. Individual patients were used as the unit of analysis. Intention to treat data from the individual clinical studies were used for the analysis. Revman 5.3 software (Cochrane Collaboration, Oxford, UK) was used for data synthesis and analysis. The random effects model for analysis was used due to the anticipated clinical between-study heterogeneity, and the results were reported in a forest plot with 95% confidence intervals (CIs).

### 2.7. Predefined Sources of Heterogeneity

Heterogeneity among the studies was assessed using the *I*^2^ statistic. We quantified inconsistency by calculating *I*^2^ and interpreted it using the following guide: 0% to 50% may represent low heterogeneity; 50% to 75% may represent moderate heterogeneity; and 75% to 100% may represent high heterogeneity.

The variables of interest included endotracheal tube or LMA. Based on the clinical assumption that this variable will lead to incidences of airway complications, we planned to separately analyze outcomes according to the type of airway device (endotracheal tube vs. LMA).

Two authors (C.-H.K., S.Y.L.) planned to construct funnel plots and evaluate their symmetry to visually assess publication bias for outcomes.

## 3. Results

### 3.1. Characteristics of Trials and Patients

**Figure 1:**
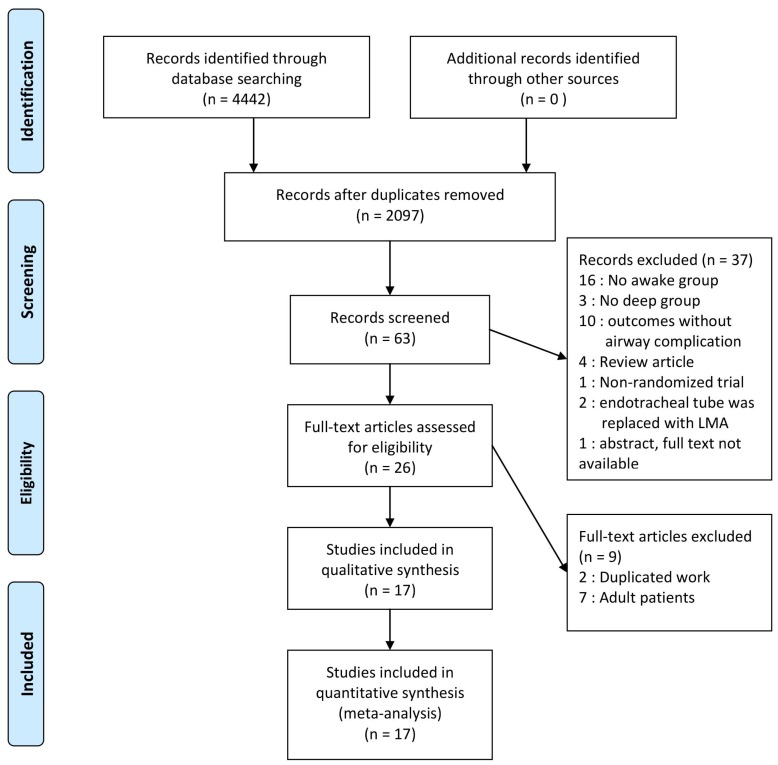
Flow diagram of the included and excluded studies.

Searches of electronic databases retrieved 4442 potentially eligible reports published up to October 2017. After retrieving the full text of the relevant studies, 2345 records were excluded for duplicated work. A total of 2034 records were excluded after initial screening; by examining titles and another 37 records were excluded because they were not RCT, did not report airway complications, did not compare between deep and awake status and used different emergence technique. Another seven trials were excluded since they were studies of adult patients. A total of 17 full-text randomized trials [7,10,13,14,15,16,17,18,19,20,21,22,23,24,25,26,27] were included in the final analysis (Figure 1).

Figure 1 represents the Preferred Reporting Items for Systematic reviews and Meta-Analyses (PRISMA) flow diagram and summarizes the reasons for exclusion of records. Data from a total of 1881 pediatric patients including 942 in the awake group and 939 in the deep group, were available for analysis. Details of the 17 trials and the baseline characteristics of the included study populations are demonstrated in Table 1. 

### 3.2. Methodological Quality and Risk of Bias (Risk of Bias Assessment)

**Figure 2:**
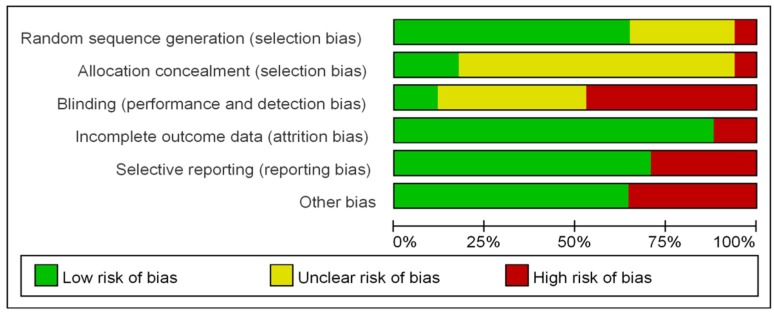
Risk of bias graph. Review author’s judgment about each risk of bias item presented as percentage across studies.

The summary and results of methodological quality assessment of the included RCTs are demonstrated graphically in Figure 2. Detailed assessments of the risk of bias for each trial are in Table S2.

In all studies, patients were randomly assigned to the groups but the method used for randomization were not described in 5/17 studies. In most studies, there were no descriptions of allocation concealment (13/17). The risk of performance and detection bias was mostly unclear (7/17) or high (8/17). Since it is definitely distinguished between deep anesthesia and wakefulness, it is difficult to blind the observer completely. The majority of trials were assigned low risk of attribution bias, reporting bias and other bias. Funnel plots for the results are shown in Supplemental Figure 2, which most outcomes appear symmetrical in shape, indicating a low risk of publication bias.

### 3.3. Outcome Synthesis

**Figure 3:**
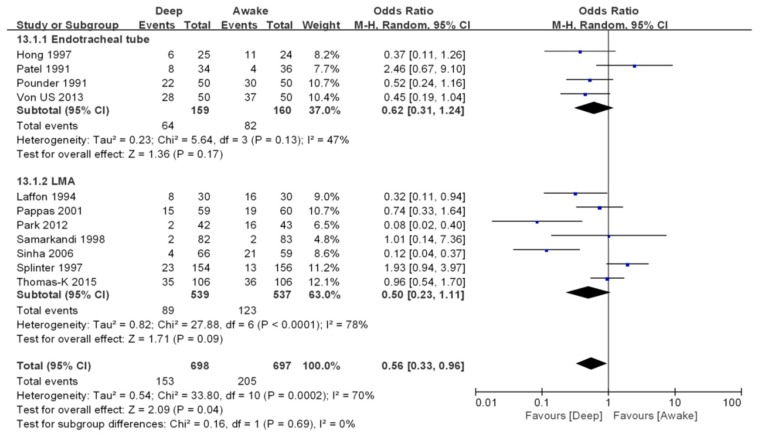
Forest plot of comparison. Deep extubation (experimental) vs. awake extubation (control). Outcome: Overall complications.

**Overall airway complications.** Overall airway complications were reported in 11 studies including 1395 patients (Figure 3) [13,14,15,17,18,19,22,23,25,26,27]. The risk of overall airway complications were lower in deep extubation group than awake extubation group (OR 0.56, 95% CI 0.33–0.96, *p* = 0.04). A moderate level of heterogeneity among the studies existed (*I*^2^ = 70%, *p* = 0.0002).

**Figure 4:**
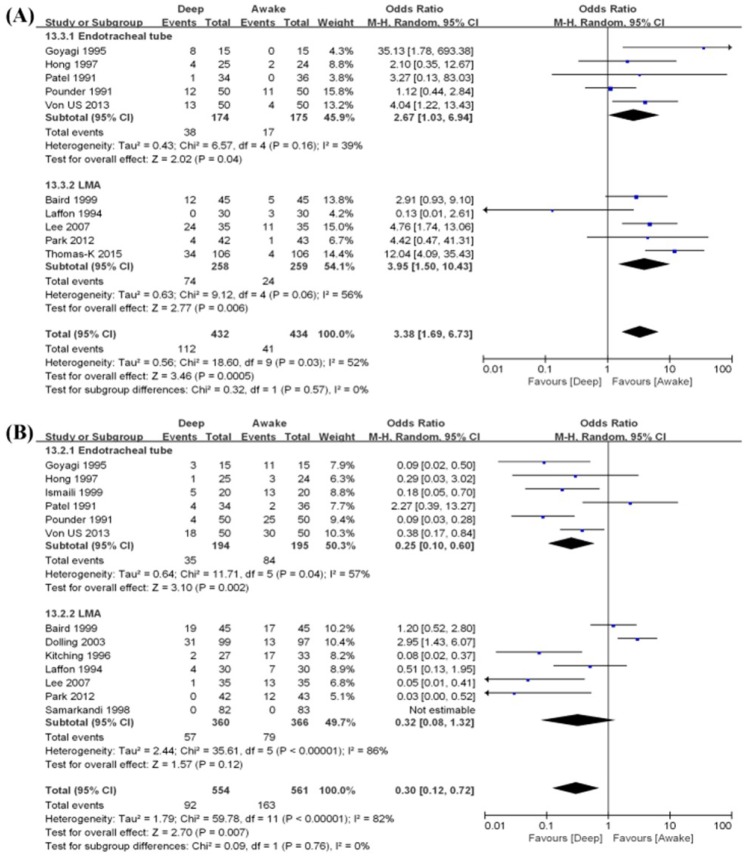

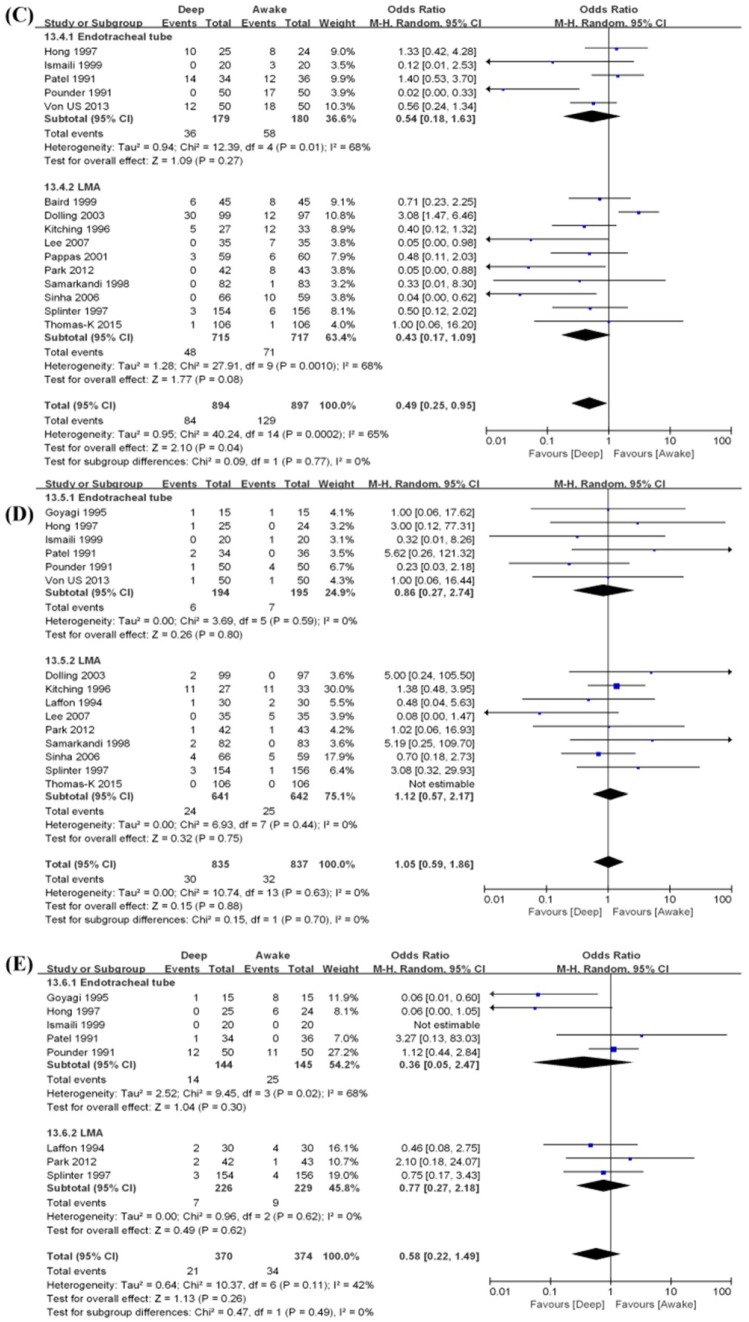
Forest plot of comparison. Deep extubation (experimental) vs. Awake extubation. (control). Outcome: (**A**) Airway obstruction, (**B**) cough, (**C**) desaturation, (**D**) laryngospasm, and (**E**) breath-holding.

**Airway obstruction.** Airway obstruction was reported in 10 studies including 866 patients (Figure 4A) [13,14,15,16,17,20,24,25,26,27]. The risk of airway obstruction was higher in deep extubation than awake extubation group (OR 3.38, 95% CI 1.69–6.73, *p* = 0.0005). A moderate level of heterogeneity among the studies existed (*I*^2^ = 52%, *p* = 0.03).

**Cough.** Cough was reported in 12 studies including 1115 patients (Figure 4B) [7,10,13,14,15,16,17,19,20,24,25,26]. The risk of cough was lower in deep extubation group than awake extubation group (OR 0.30, 95% CI 0.12–0.72, *p* = 0.007). A high level of heterogeneity among the studies existed (*I*^2^ = 82%, *p* < 0.00001).

**Desaturation (<96%).** Desaturation was reported in 15 studies including 1791 patients (Figure 4C) [7,10,13,14,17,18,19,20,21,22,23,24,25,26,27]. The risk of desaturation was lower in deep extubation group than awake extubation group (OR 0.49, 95% CI 0.25-0.95, P = 0.04). A moderate level of heterogeneity among the studies existed (*I*^2^ = 65%, *p* = 0.0002).

**Laryngospasm.** Laryngospasm was reported in 15 studies including 1672 patients (Figure 4D) [7,10,13,14,15,16,17,18,19,21,23,24,25,26,27]. No significant difference in the risk of laryngospasm between the deep and awake extubation group was found (OR 1.05 95% CI 0.59–1.86, *p* = 0.88). A low level of heterogeneity among the studies existed (*I*^2^ = 0%, *p* = 0.63).

**Breath-holding.** Breath-holding was reported in 8 studies including 744 patients (Figure 4E) [13,14,15,16,17,18,21,25]. There was no difference in risk of breath holding between the deep and awake extubation group (OR 0.58, 95% CI 0.22–1.49, *p* = 0.26). A low level of heterogeneity among the studies existed (*I*^2^ = 42%, *p* = 0.11).

### 3.4. Subgroup Analysis

#### Endotracheal Tube vs. Laryngeal Mask Airway (LMA)

**Overall airway complications.** Overall airway complications were reported in four endotracheal tube studies [13,14,17,26] including 319 patients and in seven LMA studies [15,18,19,22,23,25,27] including 1076 patients (Figure 3). For both endotracheal tube and LMA patients, no significant difference in the risk of overall airway complications between the deep and awake extubation groups was found (OR 0.62, 95% CI 0.31–1.24, *p* = 0.17 with low level of heterogeneity (*I*^2^ = 47%, *p* = 0.13) for endotracheal group; OR 0.5, 95% CI 0.23–1.11, *p* = 0.09 with a high level of heterogeneity among the studies (*I*^2^ = 78%, *p* < 0.0001) for the LMA group).

**Airway obstruction.** Airway obstruction was reported in five endotracheal tube studies [13,14,16,17,26] including 349 patients and in five LMA studies [15,20,24,25,27] including 517 patients (Figure 4A). For endotracheal tube patients, the risk of airway obstruction was higher in deep extubation group than awake extubation group (OR 2.67, 95% CI 1.03–6.94, *p* = 0.04) with a low level of heterogeneity among the studies (*I*^2^ = 39%, *p* = 0.16). For LMA patients, the risk of airway obstruction was also higher in deep extubation group than awake extubation group (OR 3.95, 95% CI 1.50–10.43, *p* = 0.006) with a moderate level of heterogeneity among the studies (*I*^2^ = 50%, *p* = 0.06)

**Cough.** Cough was reported in 6 endotracheal tube studies [13,14,16,17,21,26] including 389 patients and in seven LMA studies [7,10,15,19,20,24,25] including 726 patients (Figure 4B).

For endotracheal tube patients, the risk of cough was lower in deep extubation group than awake extubation group (OR 0.25, 95% CI 0.10–0.60, *p* = 0.002) with a moderate level of heterogeneity among the studies (*I*^2^ = 57%, *p* = 0.04). However, for LMA patients, there was no significant difference in the risk of cough between the deep and awake extubation (OR 0.32, 95% CI 0.08–1.32, *p* = 0.12) with a high level of heterogeneity among the studies (*I*^2^ = 86%, *p* < 0.00001).

**Desaturation (<96%).** Desaturation was reported in 5 endotracheal tube studies [13,14,17,21,26] including 379 patients and in 10 LMA studies [7,10,18,19,20,22,23,24,25,27] including 1432 patients (Figure 4C). For both groups, no significant difference in the risk of desaturation between the deep and awake extubation was found (OR 0.54, 95% CI 0.18–1.63, *p* = 0.27 with a moderate level of heterogeneity (*I*^2^ = 68%, *p* = 0.01) for endotracheal group; OR 0.43, 95% CI 0.17–1.09, *p* = 0.08 with a moderate level of heterogeneity among the studies (*I*^2^ = 68%, *p* = 0.001) for the LMA group.

**Laryngospasm.** Laryngospasm were reported in 6 endotracheal tube studies [13,14,16,17,21,26] including 389 patients and in 9 LMA studies [7,10,15,18,19,23,24,25,27] including 1283 pediatric patients (Figure 4D). There was no significant difference in the risk of laryngospasm between the deep and awake extubation regardless use of airway device (OR 0.86, 95% CI 0.27-2.74, P = 0.80 with a low level of heterogeneity (*I*^2^ = 0%, *p* = 0.59) for endotracheal group; OR 1.12, 95% CI 0.57-2.17, *p* = 0.75 with a low level of heterogeneity (*I*^2^ = 0%, *p* = 0.44) for LMA group).

**Breath holding.** Breath holding were reported in 5 endotracheal tube studies [13,14,16,17,21] including 289 patients and in 3 LMA studies [15,18,25] including 455 pediatric patients (Figure 4E). No significant difference in the risk of breath holding between the deep and awake extubation groups was found (OR 0.36, 95% CI 0.05-2.47, P =0.30 with a moderate level of heterogeneity among the studies (*I*^2^ = 68%, *p* = 0.02) for endotracheal group; OR 0.77, 95% CI 0.27–2.18, *p* = 0.62 with a low level of heterogeneity (*I*^2^ = 0%, *p* = 0.62) for LMA group.

## 4. Discussion

This meta-analysis included 17 randomized trials (1881 patients) that assessed airway complications between awake and deep extubation in pediatric patients after general anesthesia. It combined all data available for treatment comparisons. Meta-analysis suggests that deep extubation in pediatric patients reduced the risk of overall complications including cough and desaturation compared with awake extubation. On the other hand, the risk of airway obstruction was increased in deep extubation compared with awake extubation, and no difference was observed in the risk of laryngospasm and breath holding between awake and deep extubation.

Subgroup analyses revealed that the risk of overall airway complications was similar between the deep and awake extubation groups with respect to both endotracheal tube and LMA. Random effects models were mainly used to analyze the outcomes, due to the observed heterogeneity among the study populations and the variability in interventional treatments. Overall, a low to high level of between-study statistical heterogeneity was identified.

Pediatric patients are more irritable and sensitive to airway stimulation than adults [28]. Airway irritation may cause airway complications such as cough, laryngospasm, or excessive secretion. We found a reduction in the overall airway complications associated with deep extubation, which was discordance with the results of subgroup analysis. This finding might be explained by heterogeneity. As mentioned above, ‘overall airway complication’ is defined as ‘total or overall events’ in each study. Individual outcomes included in ‘total events’ are slightly varies among the studies. For examples, desaturation was excluded from ‘total events’ in some study [17], while laryngospasm was excluded from ‘overall events’ in another study [22]. This may explain the moderate to high level of heterogeneity of the overall airway complications.

Airway obstruction is a preventable complication that should be considered during the emergence from general anesthesia. The risk of airway obstruction was found to be higher after deep extubation than awake extubation with both LMA and endotracheal intubation since laryngeal reflex is not sufficiently recovered in deep plane of anesthesia [29]. It is consistent with the findings from previous meta-analysis [30].

Among airway complications, laryngospasm and desaturation pose life-threatening risks. The prevention of these complications is essential to improve safety and minimize risk during general anesthesia. The incidence of laryngospasm is reported to be 0.78–5% [31], but it is more frequent in children, smokers, or patients with airway infection [32]. In this study, the incidence of laryngospasm is shown to be about 3.7%, whereas several previous studies have reported that the incidence of laryngospasm is as high as 21–26% after tonsillectomy and adenoidectomy [33]. In order to prevent this complication, it was recommended to remove airway devices under deep anesthesia or virtually conscious state since laryngospasm can occur under light anesthesia [33]. In the current meta-analysis, no significant difference in the risk of laryngospasm and desaturation between the deep and awake extubation groups was found, and therefore, there is no conclusive evidence supporting one status over the other for minimizing the risk of laryngospasm in this study.

The definition of desaturation varies between studies. Most trials defined desaturation as SpO_2_ < 90% [13,14,18,21,22,24,27] or < 95% [7,10,23,25,26]. One trial reported both < 90% and < 95% separately [17]. Another trial reported the incidence of desaturation according to SpO_2_ < 91%, < 94%, and < 96% [20]. In the current study, desaturation was defined as SpO_2_ < 96% to include as much data as possible. Desaturation may occur for several reasons. As mentioned above, deep extubation may lead to airway obstruction, which disturbs sufficient ventilation and results in desaturation. However, the risk of desaturation was found to be lower after deep extubation than awake extubation, though deep extubation significantly increased airway obstruction. This may be explained by the assumption that airway obstruction can easily be noticed by anesthesiologists and managed by jaw lifting or oral airway insertion. Cough that was more common in awake extubation group, may be another reason for desaturation. Persistent cough may increase intrathoracic pressure, leading to perfusion-ventilation mismatch [25]. Tube biting also may lead to desaturation. In previous meta-analysis, tube biting was higher in awake extubation group compared with deep extubation group [30]. Tube biting makes ventilation obstructive or restrictive, which may cause excessive negative intrathoracic pressure, which could result in pulmonary edema. This finding may be consistent with the fact that the degree of wakefulness does not necessarily correlate with the occurrence of oxygen desaturation in pediatric patients [34].

Cough may irritate airway passage, and the risk of cough was lower in the deep extubation group than in the awake extubation group. This result was applied only with endotracheal tube. It is in close agreement with recent meta-analysis which reported that LMA reduces cough compared to endotracheal tube [35]. Cough is a reflex response to protect respiratory tract from irritants. Airway devices, especially endotracheal tube, can provoke cough by stimulating the larynx or trachea. Excessive cough may cause hypertension, tachycardia, increased ocular pressure, and increased intracranial pressure [36]. Furthermore, sustained cough may lead to secondary complications, such as hoarseness, postoperative bleeding, hypoxia and secretions [25,26]. Therefore, these complications might be relevant in terms of the overall complications and affect the quality of recovery. Deep extubation may be recommended for patients with intraocular surgery or cerebral aneurysm with the advantage of hemodynamic stability [37].

There are several limitations in this study. First, more than half of the included studies in the current meta-analysis were conducted and published in 1990s. The result of current clinical practice could not be reflected in this study. For example, studies using desflurane was not found. Second, it may be controversial that cough was regarded as a complication. It is a protective mechanism to protect the respiratory tract from various irritants. However, as mentioned above, excessive and sustained cough may be harmful and lead to secondary complications. Furthermore, coughs observed in the awake group were not protective responses, but rather reactions caused by airway device stimulus. Third, it would have been more relevant for the clinician if the observed complications were chosen or weighed according to the severity of complication. A brief episode of laryngospasm, bronchospasm, or desaturation could be resolved instantly whereas laryngospasm or bronchospasm leading to long episodes of hypoxia are of significant clinical implication. However, this may be possible when the available data was analyzed objectively. This meta-analysis could only analyze the presented data and categorizing presented data arbitrarily can undermine the objectivity of the study.

## 5. Conclusions

This meta-analysis demonstrated that deep extubation in pediatric patients may reduce the risk of overall airway complications including cough and desaturation compared with awake extubation. However, deep extubation may increase airway obstruction in children after general anesthesia regardless use of endotracheal tube or LMA. Therefore, deep extubation may be recommended in pediatric patients to minimize overall airway complications except airway obstruction and the clinicians may choose the way of extubation according to the risk of airway complications of pediatric patients.

## Figures and Tables

**Table 1:** Baseline charactersitics and population of the included randomized trials (*n* = 17).

Author	Year	Nomber of Patients (Deep/Awake)	Language	Age (year)		Weight (kg)		Airway Device	Anesthetics	Neuromuscular Blockade	Type of Surgery
				Deep	Awake	Deep	Awake				
Baird	1999 [20]	90 (45/45)	English	7.0	7.8	26.9	28.5	LMA	Inhalational	No	Not mentioned
Dolling	2003 [7]	196 (99/97)	English	5	5	22	21	LMA	Inhalational	No	Dental surgery
Goyagi	1995 [16]	30 (15/15)	Japanese	5.2	5.6	21.7	22.1	ETT	Inhalational	No	T/A, ventilation tube, or herniotomy
Hong	1997 [17]	49 (25/24)	Korean	6.4	5.8	22.4	20.8	ETT	Inhalational	pancuronium	T/A
Ismaili	1999 [21]	40 (20/20)	French	5.9	5.7	22.1	22.6	ETT	Inhalational	No or vecuronium	Ophthalmic surgery
Kitching	1996 [9]	60 (27/33)	English	Not mentioned		Not mentioned		LMA	Inhalational	No	Urogenital or lower limb plastic surgery
Laffon	1994 [15]	60 (30/30)	English	0.5	0.5	18	19	LMA	Inhalational	No	Minor urologic or lower abdominal surgery
Lee	2007 [24]	70 (35/35)	English	4	3	16.8	17.8	LMA	Inhalational	No	Urologic, orthopedic or plastic surgery
Pappas	2001 [22]	119 (59/60)	English	3.3	2.9	21.5	15	LMA	Inhalational	No	Infra-umbilical surgery
Park	2012 [25]	85 (42/43)	English	4.2	3.9	16.2	15.9	LMA	Inhalational	No	Inguinal hernia repair or hydrocelectomy
Patel	1991 [13]	70 (34/36)	English	4.7	4.0	18.4	17.0	ETT	Inhalational	succinylcholine	Strabismus or T/A
Pounder	1991 [14]	100 (50/50)	English	2.4	2.2	13.5	13	ETT	Inhalational	succinylcholine	Minor urologic or abdominal herniotomy
Samarkandi	1998 [19]	165 (82/83)	English	3.7	3.6	15.6	15.4	LMA	Inhalational	No	Lower limb or perineal surgery
Sinha	2006 [23]	125 (66/59)	English	2.6	2.7	10.6	11.0	LMA	Inhalational	No	Herniotomy, orchiopexy, or lower limb plastic surgery
Splinter	1997 [18]	310 (154/156)	English	6.1	6.9	24	28	LMA	Inhalational	No	Not mentioned
Thomas-K	2015 [27]	212 (106/106)	English	7.7	6.8	Not mentioned (Only BMI)		LMA	Inhalational	No	Pediatric, orthopedic, ophthalmic or plastic surgery
Von US	2013 [26]	100 (50/50)	English	4	5	18	20	ETT	Inhalational	Yes, but not mentioned which was used	T/A

Age and weight are expressed as the mean. LMA = Laryngeal mask; ETT = Endotracheal tube; T/A = Tonsillectomy and adenoidectomy.

## Supplementary Materials

The following are available online at http://www.mdpi.com/2077-0383/7/10/353/s1; Figure S1: Risk of bias summary, Review author’s judgment about each risk of bias item for each included study; Figure S2: Funnel plot of comparison, deep extubation (experimental) vs. awake extubation (control) outcome: (A) overall complications, (B) airway obstruction, (C) cough, (D) desaturation, (E) laryngospasm, (F) breath-holding; Table S1. Search strategy for each database; Table S2: Details for judgment for each risk of bias.
