# Supplementary material for: Deep vs. Awake Extubation and LMA Removal in Terms of Airway Complications in Pediatric Patients Undergoing Anesthesia: A Systemic Review and Meta-Analysis
Source: J Clin Med. 2018 Oct 14;7(10):353. doi: 10.3390/jcm7100353 (PMC6210687; doi:10.3390/jcm7100353)
Supplement: Supplementary file 1 [file jcm-07-00353-s001.zip › Suppl Table 2.docx]

Supplemental Table 2. Details for judgement for each risk of bias.

| Study | Bias | Author’s judgement | Reason for judgement |
| --- | --- | --- | --- |
| Baird 1999 | Random sequence generation  (selection bias) | Unclear | There is no description of method used for random sequence generation |
|  | Allocation concealment  (selection bias) | Unclear | There is no description whether envelops were opaque. |
|  | Blinding  (performance and detection bias) | High | “It was not possible to double-blinded the study, as observers needed to be able to see patients’ airways.” |
|  | Incomplete outcome data  (attrition bias) | Low | Outcomes are reported for 300 patients |
|  | Selective reporting  (reporting bias) | Low | All pre-specified and expected outcomes are reported |
|  | Other bias | Low | No other bias was detected. |
| Dolling 2003 | Random sequence generation  (selection bias) | Low | By the toss of a coin |
|  | Allocation concealment  (selection bias) | Unclear | There is no description of allocation concealment |
|  | Blinding  (performance and detection bias) | High | “The anaesthetist was informed of the assigned group before surgery was completed.” |
|  | Incomplete outcome data  (attrition bias) | Low | Outcomes are reported for available cases. |
|  | Selective reporting  (reporting bias) | Low | All pre-specified and expected outcomes are reported |
|  | Other bias | Low | No other bias was detected |
| Goyagi 1995 | Random sequence generation  (selection bias) | Unclear | There is no description of method used for random sequence generation. |
|  | Allocation concealment  (selection bias) | Unclear | There is no description of allocation concealment |
|  | Blinding  (performance and detection bias) | Unclear | There is no description of blinding. |
|  | Incomplete outcome data  (attrition bias) | Low | Outcomes are reported for 30 patients |
|  | Selective reporting  (reporting bias) | Low | All pre-specified and expected outcomes are reported. |
|  | Other bias | Low | No other bias was detected. |
| Hong 1997 | Random sequence generation  (selection bias) | Low | Drawing lots |
|  | Allocation concealment  (selection bias) | Unclear | There is no description of allocation concealment |
|  | Blinding  (performance and detection bias) | High | It was not blinded. |
|  | Incomplete outcome data  (attrition bias) | Low | Outcomes are reported for 49 patients. |
|  | Selective reporting  (reporting bias) | Low | All pre-specified and expected outcomes are reported. |
|  | Other bias | Low | No other bias was detected. |
| Ismaili 1999 | Random sequence generation  (selection bias) | Unclear | There is no description of method used for random sequence generation. |
|  | Allocation concealment  (selection bias) | Unclear | There is no description of allocation concealment |
|  | Blinding  (performance and detection bias) | Unclear | There is no description of blindness. |
|  | Incomplete outcome data  (attrition bias) | Low | Outcomes are reported for 40 patients |
|  | Selective reporting  (reporting bias) | High | Outcome of SpO2 is not reported. |
|  | Other bias | Low | No other bias was detected. |
| Kitching 1996 | Random sequence generation  (selection bias) | Low | “By tossing a coin” |
|  | Allocation concealment  (selection bias) | Unclear | There is no description of allocation concealment |
|  | Blinding  (performance and detection bias) | Unclear | “single-blind study” |
|  | Incomplete outcome data  (attrition bias) | Low | Outcomes are reported for 60 patients |
|  | Selective reporting  (reporting bias) | High | Outcome of airway obstruction was not reported. |
|  | Other bias | High | There is a difference in sample size between two groups. |
| Laffon 1994 | Random sequence generation  (selection bias) | Unclear | There is no description of method used for random sequence generation. |
|  | Allocation concealment  (selection bias) | Unclear | There is no description of allocation concealment |
|  | Blinding  (performance and detection bias) | Unclear | There is no description of blindness. |
|  | Incomplete outcome data  (attrition bias) | Low | Outcomes are reported for 60 patients. |
|  | Selective reporting  (reporting bias) | Low | All pre-specified and expected outcomes are reported. |
|  | Other bias | Low | No other bias was detected. |
| Lee 2007 | Random sequence generation  (selection bias) | Unclear | There is no description of method used for random sequence generation. |
|  | Allocation concealment  (selection bias) | Unclear | There is no description of allocation concealment |
|  | Blinding  (performance and detection bias) | High | “AS deep or awake patients could be clearly distinguished from one another, the observers could not be blinded to the technique of removal of LT.” |
|  | Incomplete outcome data  (attrition bias) | Low | Outcomes are reported for 70 patients. |
|  | Selective reporting  (reporting bias) | Low | All pre-specified and expected outcomes are reported. |
|  | Other bias | Low | No other bias was detected. |
| Pappas 2001 | Random sequence generation  (selection bias) | Low | “using a computer-generated random sequence” |
|  | Allocation concealment  (selection bias) | Unclear | There is no description of allocation concealment |
|  | Blinding  (performance and detection bias) | Low | “a research nurse, who was blinded to the anesthetic drug and technique used, recorded all study variables. |
|  | Incomplete outcome data  (attrition bias) | Low | Outcomes are reported for available data. |
|  | Selective reporting  (reporting bias) | Low | All pre-specified and expected outcomes are reported. |
|  | Other bias | High | “One of the limitations of this study is that it was terminated prematurely due to the occurrence of critical events in four children in Group 1.” |
| Park 2012 | Random sequence generation  (selection bias) | Low | “using a computer-generated random numbers table” |
|  | Allocation concealment  (selection bias) | High | “Three practitioners conducted this study, one of whom was aware of group allocation and was responsible for anesthesia emergence and LMA removal in each group” |
|  | Blinding  (performance and detection bias) | High | “the observer was not truly blinded because the anesthetic state and awake state were obviously different.” |
|  | Incomplete outcome data  (attrition bias) | High | This study included 92 patients but reported 85 patients without any reasons for exclusion. |
|  | Selective reporting  (reporting bias) | Low | All pre-specified and expected outcomes are reported. |
|  | Other bias | Low | No other bias was detected. |
| Patel 1991 | Random sequence generation  (selection bias) | Low | “patients were randomly assigned to one of the two groups by opening a sequentially numbered sealed envelope.” |
|  | Allocation concealment  (selection bias) | Unclear | There is no description whether envelops were opaque. |
|  | Blinding  (performance and detection bias) | Low | “In the postanesthetic period, the patients were evaluated for signs and symptoms of croup after extubation by a blinded observer” |
|  | Incomplete outcome data  (attrition bias) | Low | Outcomes are reported for 70 patients |
|  | Selective reporting  (reporting bias) | Low | All pre-specified and expected outcomes are reported. |
|  | Other bias | High | “One obvious bias of this study is that patients in group 1 received 100% oxygen for 5 min before tracheal extubation, whereas patients in group 2 were maintained on 50% nitrous oxide until extubation and were given 100% oxygen for 5 min after extubation.” |
| Pounder 1991 | Random sequence generation  (selection bias) | High | It was randomized only for volatile agents. |
|  | Allocation concealment  (selection bias) | Unclear | There is no description of allocation concealment |
|  | Blinding  (performance and detection bias) | Unclear | There is no description of blindness for deep or awake. |
|  | Incomplete outcome data  (attrition bias) | Low | Outcomes are reported for 100 patients |
|  | Selective reporting  (reporting bias) | Low | All pre-specified and expected outcomes are reported. |
|  | Other bias | Low | No other bias was detected. |
| Samarkandi 1998 | Random sequence generation  (selection bias) | Low | “according to random number generated by computer” |
|  | Allocation concealment  (selection bias) | Unclear | There is no description of allocation concealment |
|  | Blinding  (performance and detection bias) | Unclear | There is no description of blindness. |
|  | Incomplete outcome data  (attrition bias) | Low | Outcomes are reported for 165 patients |
|  | Selective reporting  (reporting bias) | High | Outcome of bronchospasm was not reported. |
|  | Other bias | Low | No other bias was detected. |
| Sinha 2006 | Random sequence generation  (selection bias) | Low | “using computer-generated random numbers from sealed opaque envelops” |
|  | Allocation concealment  (selection bias) | Low | “sealed opaque envelops” |
|  | Blinding  (performance and detection bias) | High | It was not blinded |
|  | Incomplete outcome data  (attrition bias) | Low | Outcomes are reported for available data. |
|  | Selective reporting  (reporting bias) | High | Outcomes such as clenching teeth and biting were not reported. |
|  | Other bias | High | There is a difference in sample size between two groups. |
| Splinter 1997 | Random sequence generation  (selection bias) | Low | “by computer-generated random numbers table” |
|  | Allocation concealment  (selection bias) | Unclear | There is no description of allocation concealment |
|  | Blinding  (performance and detection bias) | High | It was not blinded. |
|  | Incomplete outcome data  (attrition bias) | High | 333 patients were enrolled, 25 patients were excluded and outcomes are reported for only 310 patients. |
|  | Selective reporting  (reporting bias) | High | Outcome of biting was not reported.  Retching and vomiting was reported as summated. |
|  | Other bias | Low | No other bias was detected |
| Thomas-K 2015 | Random sequence generation  (selection bias) | Low | “Randomisation was achieved by the use of random numbers (generated in advance by computer)” |
|  | Allocation concealment  (selection bias) | Low | “Sealed envelopes detailing the allocation for each recruit and the relevant data forms were stored securely in the theatre department: |
|  | Blinding  (performance and detection bias) | Unclear | There is no description of blindness. |
|  | Incomplete outcome data  (attrition bias) | Low | Outcomes are reported for available data. |
|  | Selective reporting  (reporting bias) | Low | All pre-specified and expected outcomes are reported. |
|  | Other bias | High | “A potential source of bias, however, is that any immediate complications occurring after LMA removal in anaesthetized patients were recorded by the anaesthetist, whereas all other observations were made by nurses in the PACU.” |
| Von US 2013 | Random sequence generation  (selection bias) | Low | “computer-generated block randomization” |
|  | Allocation concealment  (selection bias) | Low | “concealed in a closed envelop” |
|  | Blinding  (performance and detection bias) | High | “attending anaesthesiologist and data recorder in theatre were then aware of the study arm” |
|  | Incomplete outcome data  (attrition bias) | Low | Outcomes are reported for 100 patients. |
|  | Selective reporting  (reporting bias) | Low | All pre-specified and expected outcomes are reported. |
|  | Other bias | High | “the children in deep group demonstrated a tendency towards more severe grade of OSAS.” |
