# Supplementary material for: Deep vs. Awake Extubation and LMA Removal in Terms of Airway Complications in Pediatric Patients Undergoing Anesthesia: A Systemic Review and Meta-Analysis
Source: J Clin Med. 2018 Oct 14;7(10):353. doi: 10.3390/jcm7100353 (PMC6210687; doi:10.3390/jcm7100353)
Supplement: Supplementary file 1 [file jcm-07-00353-s001.zip › Suppl Table 1.docx]

Supplemental Table 1. Search strategy for each database.

| Database | Order | Keywords | Results |
| --- | --- | --- | --- |
| MEDLINE | #1 | Anesthesia[mh] | 178692 |
|  | #2 | Anesthesia*[tiab] | 149924 |
|  | #3 | #1 OR #2 | 248848 |
|  | #4 | “Laryngeal masks”[mh] OR “intubation, intratracheal”[mh] OR “airway extubation”[mh] | 35972 |
|  | #5 | intubation[tiab] OR extubation[tiab] OR remov*[tiab] | 590258 |
|  | #6 | Laryngeal[tiab] AND mask*[tiab] | 4772 |
|  | #7 | #4 OR #5 OR #6 | 609219 |
|  | #8 | #3 AND #7 | 25646 |
|  | #9 | deep*[tiab] OR awake[tiab] OR anesthetized[tiab] | 310587 |
|  | #10 | #8 AND #9 | 2639 |
|  | #11 | #10 AND HSSS | 549 |
| EMBASE | #1 | anesthesia/exp | 320006 |
|  | #2 | anesthesia*:ab,ti | 182675 |
|  | #3 | #1 OR #2 | 379879 |
|  | #4 | ‘laryngeal mask’/de OR ‘endotracheal intubation’/de OR extubation/de | 72323 |
|  | #5 | Intubation:ab,ti OR extubation:ab,ti OR remov*:ab,ti | 744922 |
|  | #6 | Laryngeal:ab,ti AND mask*:ab,ti | 5853 |
|  | #7 | #4 OR #5 OR #6 | 3 and 7 |
|  | #8 | #3 AND #7 | 43852 |
|  | #9 | deep*:ab,ti OR awake:ab,ti OR anesthetized:ab,ti | 397841 |
|  | #10 | #8 AND #9 | 4368 |
|  | #11 | 10 AND RCT filter | 982 |
|  | #12 | 11 AND [embase]/lim | 886 |
| CENTRAL | #1 | [mh anesthesia] | 17792 |
|  | #2 | anesthesia*:ti,ab,kw | 38127 |
|  | #3 | #1 OR #2 | 40164 |
|  | #4 | [mh “laryngeal masks”] OR [mh “intubation, intratracheal”] OR [mh “airway extubation”] | 4149 |
|  | #5 | Intubation:ti,ab,kw OR extubation:ti,ab,kw OR remov*:ti,ab,kw | 31746 |
|  | #6 | Laryngeal:ti,ab,kw AND mask*:ti,ab,kw | 2035 |
|  | #7 | #4 OR #5 OR #6 | 32800 |
|  | #8 | #3 AND #7 | 7789 |
|  | #9 | deep*:ti,ab,kw OR awake:ti,ab,kw OR anesthetized:ti,ab,kw | 15247 |
|  | #10 | #8 AND #9 | 789 |
|  | #11 | #10 AND Trials | 785 |
| CINHAL | S1 | MH(anesthesia+) | 10674 |
|  | S2 | TI(anesthesia*) | 2586 |
|  | S3 | S1 OR S2 | 11628 |
|  | S4 | MH(laryngeal masks+) OR MH(intubation, intratracheal+) OR MH(extubation) | 4413 |
|  | S5 | TI(intubation OR extubation OR remov*) OR AB(intubation OR extubation OR remov*) | 20581 |
|  | S6 | TI(Laryngeal) OR AB(Laryngeal) | 3403 |
|  | S7 | TI(mask) OR AB(mask) | 2016 |
|  | S8 | S6 AND S7 | 555 |
|  | S9 | S4 OR S5 OR S8 | 23171 |
|  | S10 | S3 AND S9 | 1117 |
|  | S11 | TI(deep OR awake OR anesthetized) OR AB(deep OR awake OR anesthetized) | 10310 |
|  | S12 | S10 AND S11 | 83 |
|  | S13 | random* OR blind* OR allocat* OR assign* OR trial* OR placebo* OR crossover OR cross-over* OR “control group” OR groups OR intervention* |  |
|  | S14 | S12 AND S13 | 26 |
| SCOPUS | #1 | INDEXTERMS(anesthesia) | 282217 |
|  | #2 | TITLE-ABS(anesthesia*) | 250169 |
|  | #3 | #1 OR #2 | 385937 |
|  | #4 | INDEXTERMS(laryngeal masks) OR INDEXTERMS(intubation, intratracheal) OR INDEXTERMS(airway extubation) | 37810 |
|  | #5 | TITLE-ABS(intubation) OR TITLE-ABS(extubation) OR TITLE-ABS(remov*) | 1361652 |
|  | #6 | Laryngeal[tiab] AND mask*[tiab] → TITLE-ABS(laryngeal) AND TITLE-ABS(mask*) | 5483 |
|  | #7 | #4 OR #5 OR #6 | 1381911 |
|  | #8 | #3 AND #7 | 41458 |
|  | #9 | TITLA-ABS(deep*) OR TITLE-ABS(awake) OR TITLE-ABS(anesthetized) | 937601 |
|  | #10 | #8 AND #9 | 3996 |
|  | #11 | (INDEXTERMS(randomized controlled trial) OR INDEXTERMS(controlled clinical trial) OR TITLE-ABS(randomized) OR TITLE-ABS(placebo) OR INDEXTERMS(drug therapy) OR TITLE-ABS(randomly) OR TITLE-ABS(trial) OR TITLE-ABS(groups)) AND NOT (INDEXTERMS(animals) AND NOT INDEXTERMS(humans)) |  |
|  | #12 | 10 and 11 | 1238 |
| Web of Science | #1 | TS=(anesthesia*) | 133527 |
|  | #2 | TS=(laryngeal OR intubation OR extubation OR remov* OR mask*) | 964864 |
|  | #3 | #1 AND #2 | 16370 |
|  | #4 | TS=(deep* OR awake OR anesthetized) | 606832 |
|  | #5 | #3 AND #4 | 2342 |
|  | #6 | random* OR blind* OR allocat* OR assign* OR trial* OR placebo* OR crossover OR cross-over* OR intervention* |  |
|  | #7 | #5 AND #6 | 953 |
| KoreaMed | #1 | awake [ALL] extubation [ALL] or deep [ALL] extubation [ALL] or anesthetized [ALL] extubation [ALL] or awake [ALL] removal [ALL] or deep [ALL] removal [ALL] or anesthetized [ALL] removal [ALL] | 5 |
