# Supplementary figures and images for: Deep vs. Awake Extubation and LMA Removal in Terms of Airway Complications in Pediatric Patients Undergoing Anesthesia: A Systemic Review and Meta-Analysis
Source: J Clin Med. 2018 Oct 14;7(10):353. doi: 10.3390/jcm7100353 (PMC6210687; doi:10.3390/jcm7100353)

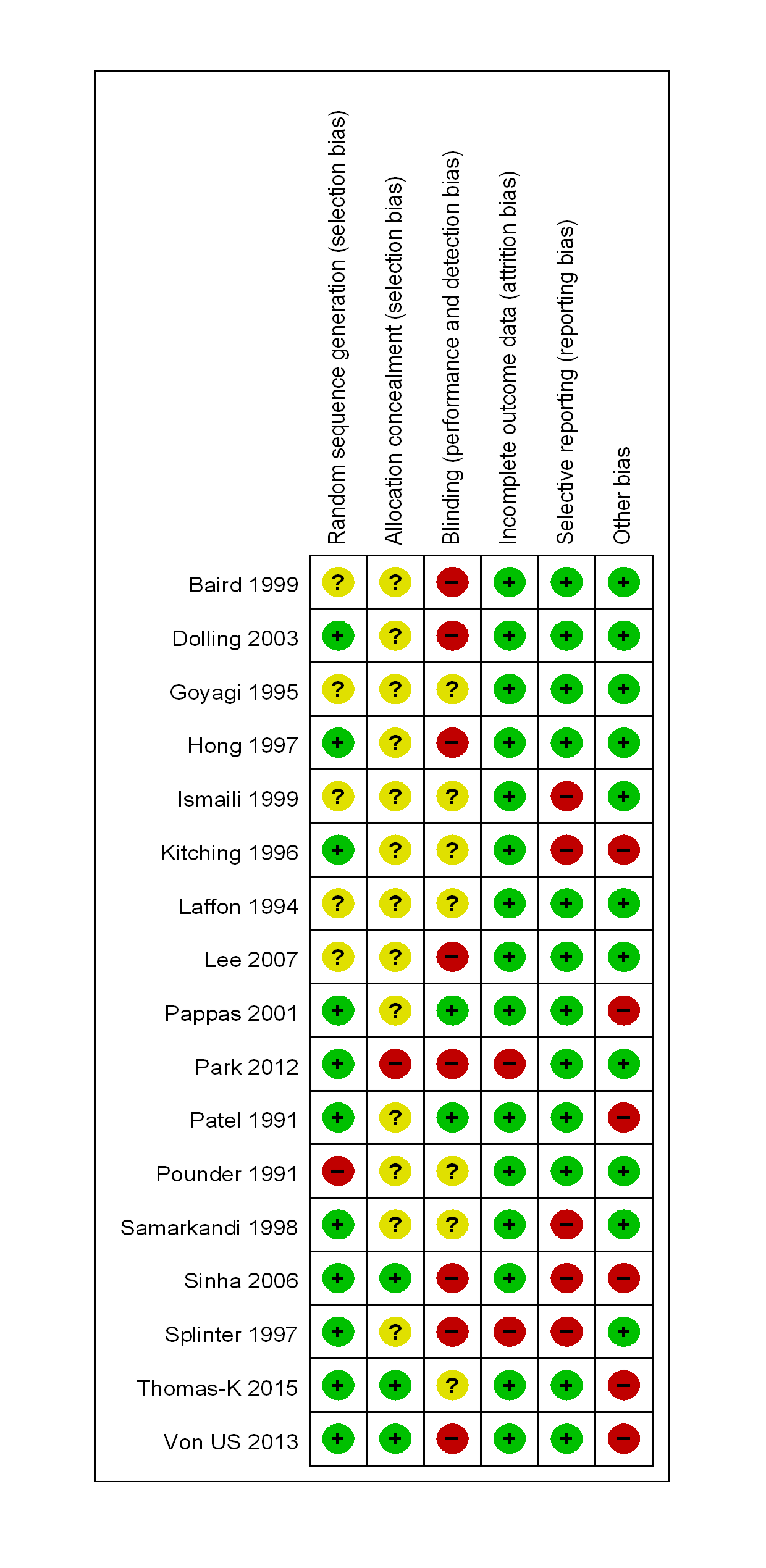

Supplement: Supplementary file 1 [file jcm-07-00353-s001.zip › Suppl Figure 1.tif]

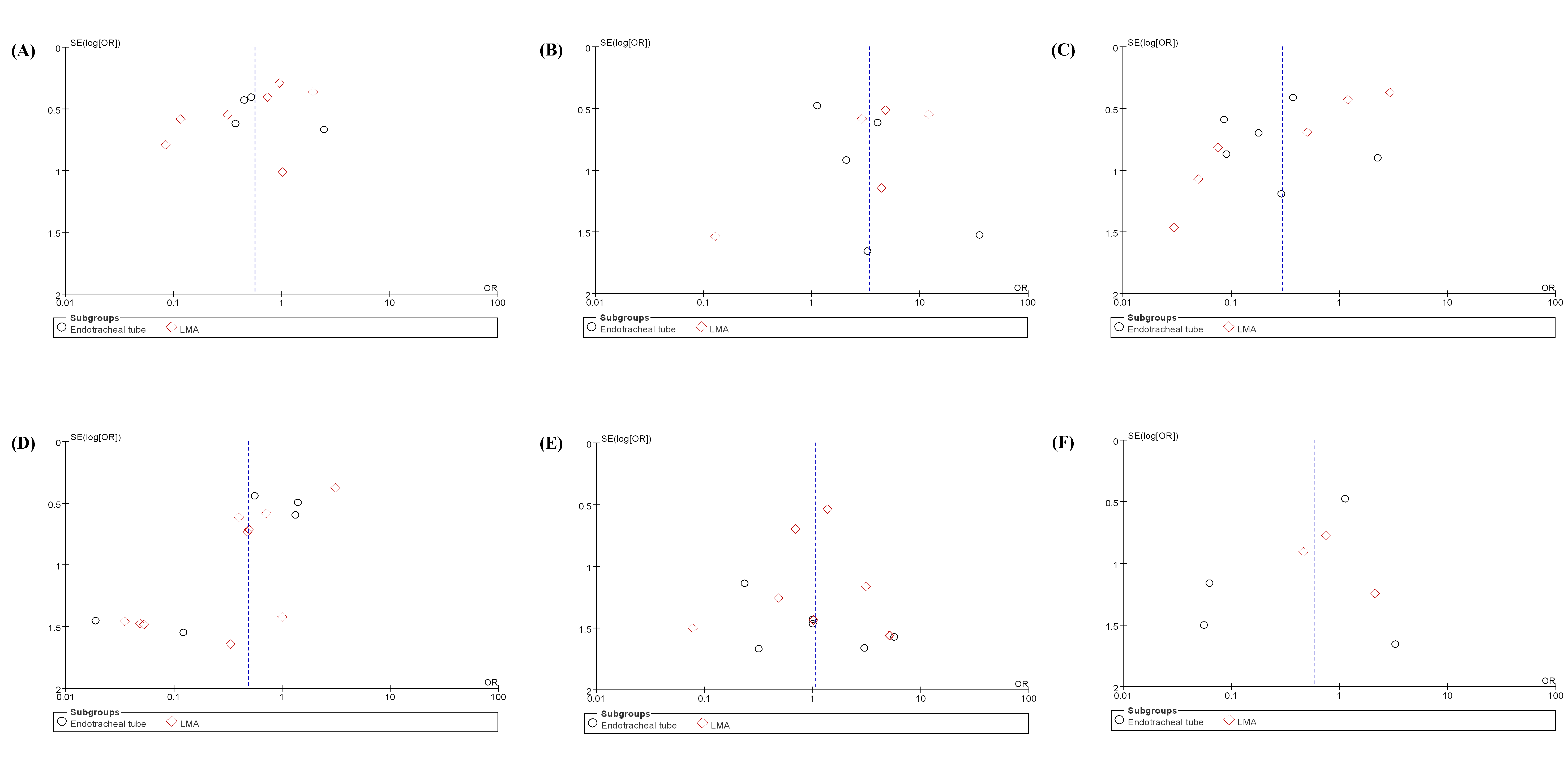

Supplement: Supplementary file 1 [file jcm-07-00353-s001.zip › Suppl Figure 2.tif]
